# Poor motor function is associated with reduced sensory processing after stroke

**DOI:** 10.1007/s00221-015-4206-z

**Published:** 2015-02-05

**Authors:** S. Floor Campfens, Sarah B. Zandvliet, Carel G. M. Meskers, Alfred C. Schouten, Michel J. A. M. van Putten, Herman van der Kooij

**Affiliations:** 1 Department of Biomechanical Engineering, MIRA Institute of Biomedical Engineering and Technical Medicine, University of Twente, PO Box 217, 7500 AE Enschede, The Netherlands; 2 Clinical Neurophysiology, MIRA Institute of Biomedical Engineering and Technical Medicine, University of Twente, Enschede, The Netherlands; 3 Department of Rehabilitation Medicine, Leiden University Medical Center, Leiden, The Netherlands; 4 Department of Biomechanical Engineering, Delft University of Technology, Delft, The Netherlands

**Keywords:** Stroke, Coherence, Afferent pathways, Motor control, Joint position perturbation, EEG

## Abstract

The possibility to regain motor function after stroke depends on the intactness of motor and sensory pathways. In this study, we evaluated afferent sensory pathway information transfer and processing after stroke with the coherence between cortical activity and a position perturbation (position-cortical coherence, PCC). Eleven subacute stroke survivors participated in this study. Subjects performed a motor task with the affected and non-affected arm while continuous wrist position perturbations were applied. Cortical activity was measured using EEG. PCC was calculated between position perturbation and EEG at the contralateral and ipsilateral sensorimotor area. The presence of PCC was quantified as the number of frequencies where PCC is larger than zero across the sensorimotor area. All subjects showed significant contralateral PCC in affected and non-affected wrist tasks. Subjects with poor motor function had a reduced presence of contralateral PCC compared with subjects with good motor function in the affected wrist tasks. Amplitude of significant PCC did not differ between subjects with good and poor motor function. Our results show that poor motor function is associated with reduced sensory pathway information transfer and processing in subacute stroke subjects. Position-cortical coherence may provide additional insight into mechanisms of recovery of motor function after stroke.

## Introduction

Stroke is a leading cause of adult-onset disability in the Western world. Rehabilitation after stroke has a strong emphasis on reducing motor impairment to improve the quality of life (Kwakkel et al. 2004). Within rehabilitation practice, sensory impairment does not receive as much attention as motor impairment does, although it is known that sensory impairment is common after stroke (Connell et al. 2008) and related to motor impairment (Schabrun and Hillier 2009).

The relation between sensory and motor impairment is unsurprising because motor control requires bidirectional interaction between cortex and periphery (Scott 2004; Baker 2007). Sensory feedback via the afferent pathways is necessary to generate proper motor commands which reach the muscles via the efferent pathways. Invasive recordings in monkeys showed that both sensory and motor cortical neural populations synchronise their oscillatory activity to peripheral signals (Williams et al. 2009). This synchronisation is thought to play an important role in the transmission of information, i.e. connectivity, within closed loop motor control (Varela et al. 2001; Fries 2005; Baker 2007). Coherence between cortical activity and muscle activity, corticomuscular coherence (CMC) (Conway et al. 1995; Halliday et al. 1998; Mima et al. 2000), is used as a measure of cortical sensorimotor integration during a motor task and depends on the information transfer across both efferent and afferent pathways (Mima et al. 2001; Pohja and Salenius 2003; Riddle and Baker 2005; Witham et al. 2011).

As a measure of both efferent and afferent pathway connectivity, changes in CMC cannot be related to changes in sensory or motor pathways. In addition, measurement of CMC requires a measurable EMG signal and thus is only possible in subjects that are able to voluntarily generate muscle force. When studying sensory and motor function after stroke with CMC, no information can be obtained from individuals without voluntary muscle control. Finally, a large downside for the potential clinical application of CMC is that it cannot be detected in all cases: even healthy subjects, with normal voluntary motor control, do not all present CMC (Ushiyama et al. 2011; Mendez-Balbuena et al. 2011; Campfens et al. 2013). The inter-individual difference in the presence of CMC reflects physiological inter-individual differences in the strength of the oscillatory corticomuscular coupling and is not the result of technical aspects such as the (mis-) placement of EEG electrodes (Ushiyama et al. 2011).

We previously showed that adding a small continuous position perturbation during an isotonic force task elicits CMC and coherence between the position perturbation and the EEG: position-cortical coherence (PCC) (Campfens et al. 2013). Position-cortical coherence represents the unidirectional information transfer across the afferent pathways because the perturbation acts as an external excitation signal for the proprioceptive system (primarily the Golgi tendon organs and muscle spindles). Coherence between position perturbation and cortical activity can only occur when sensory feedback related to the perturbation reaches the cortex via the afferent pathways: information transfer. Possibly, also neural populations that are involved in the processing and sensorimotor integration of this information synchronise their activity to the perturbation as well and contribute to PCC. Position-cortical coherence thus represents sensory information transfer and processing. The origin of PCC is comparable to the origin of steady-state evoked potentials of the visual or auditory system (Herrmann 2001). Because PCC is present in all subjects, it is a more reliable measure to study the cortical involvement in motor control than CMC (Campfens et al. 2013). In addition, as PCC represents unidirectional connectivity it has a simpler interpretation than CMC which represents bidirectional connectivity and does not allow estimation of pathway-specific properties (Campfens et al. 2014).

The aim of this study is to show the potential value of PCC as a measure of sensory pathway information transfer and processing after stroke. Based on the association between sensory and motor impairment after stroke (Schabrun and Hillier 2009), we hypothesise that stroke survivors with poor motor function have a lower PCC. We made a distinction between the presence of PCC (i.e. the number of frequencies and electrodes with significant PCC) and the mean amplitude of significant PCC in order to assess which is most informative of sensory pathway information transfer and processing.

In addition, we introduce a lateralisation index of PCC to evaluate the distribution of PCC between the lesioned and non-lesioned hemisphere. While in normal subjects PCC was localised at the contra-SM (Campfens et al. 2013), it has been shown with fMRI and EEG that stroke survivors recruit additional, ipsilateral areas during movement (Ward 2003; Serrien et al. 2004). We therefore hypothesise that stroke survivors present PCC in both hemispheres.

## Methods

Eleven first-ever hemispheric stroke survivors participated in the study (one woman). Details of the subjects are presented in Table 1; all subjects had ischaemic stroke. Subjects were either outpatients or recruited in the acute phase from the hospital wards of the Leiden University Medical Center. Subjects were in the subacute phase after stroke (within 6 months post-stroke). The group of subjects had a dichotomous distribution on the Brunström Fugl-Meyer upper extremity (FM-UE) scale (Fugl-Meyer et al. 1975). Six subjects had a FM-UE of 55 points or higher (the maximum possible score is 66 points), these subjects were considered to have a good motor function (group: good function). A score of 55 or higher indicates that these subjects were able to move outside of synergistic movement patterns. Some of these patients still experienced loss of strength and coordination, indicated by a non-maximal score; however, they were able to perform all movements and withstand a minimal resistance during the test. The other five subjects had considerably lower FM-UE scores, these subjects all scored <20 points and were considered to have poor motor function (group: poor function). Sensory function of subjects was evaluated using the Erasmus modification of the Nottingham Sensory Assessment (EmNSA) scale (Stolk-Hornsveld et al. 2006). This scale evaluates different test items across multiple locations on the upper extremity. Test items are as follows: light touch, pressure, pin prick, sharp–blunt discrimination, and proprioception. When a subject scored less than the maximal score for a test item on more than one location on the upper extremity, the test item is marked as reduced.

**Table 1:** Overview of subjects

Subject code	Age	Group	Lesion	Days since lesion	FM-UE	AS wrist	Sensory function (EmNSA)	Task affected arm^a^
C.01	77	Good function	MCA left	106	65	0	Normal	Active
C.02	58	Good function	BG right	36	63	0	Normal	Active
C.06	77	Good function	MCA right	18	56	0	Normal	Active
C.08	35	Good function	Thal. right	22	63	0	Normal	Active
C.09	54	Good function	MCA left	60	65	0	Normal	Active
C.11	72	Good function	MCA right	13	65	0	Normal	Active
C.04	62	Poor function	MCA right	27	17	1	Normal	Relax
C.05	59	Poor function	MCA right	21	15	1+ (flex)	Normal	Relax
C.07	58	Poor function	MCA right	11	19	0	Reduced tactile	Relax
C.10	46	Poor function	MCA right	34	6	1+ (flex)	Normal	Relax
C.13	67	Poor function	MCA right	121	6	4 (flex)	Reduced tactile and proprioception	Relax

*MCA* middle cerebral artery, *BG* basal ganglia, *Thal.* thalamus, *FM-UE* Brunnström Fugl-Meyer upper extremity score (maximum 66 points), *AS* Ashworth scale, *EmNSA* Erasmus modification of the Nottingham Sensory Assessment scale

^a^All subjects performed the active task with the unaffected arm

All measurements were taken in accordance with the *Declaration of Helsinki* and were approved by the Medical Ethics review Committee of the Leiden University Medical Center (Leiden, the Netherlands). All participants gave signed informed consent before the measurements.

### Experimental set-up

**Fig. 1:**
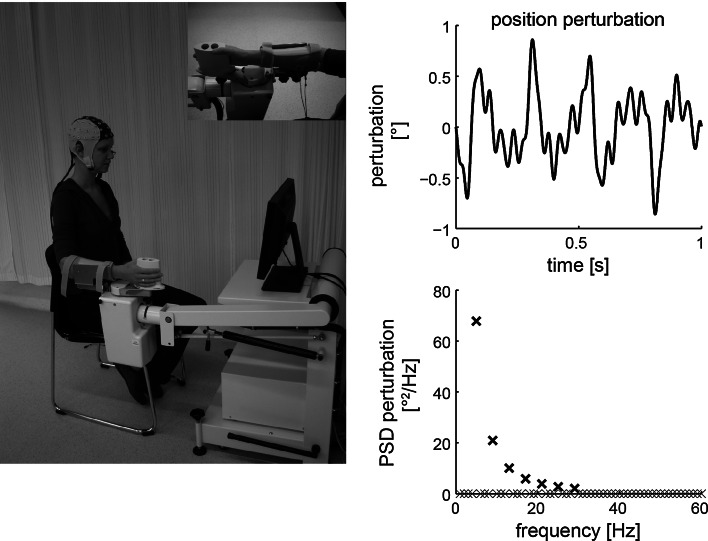
Overview of the experimental set-up (*left*) and a 1-s segment of the position perturbation (*right*). The subject holds the handle of the wrist manipulator (WM), and the *lower arm* is strapped in an arm rest using Velcro straps. To support the subject in maintaining a steady isotonic wrist flexion torque, visual feedback of the target torque and the exerted (2-Hz low-pass filtered with third-order Butterworth filter) was provided on the display in front of the subject. The position perturbation is a sum of sines with a decreasing value of the power with frequency. *PSD* power spectral density

Subjects were seated next to a wrist manipulator (Moog Inc., Nieuw-Vennep, the Netherlands), see Fig. 1. The wrist manipulator (WM) is an actuated rotating device with a single degree of freedom that can impose flexion and extension movements on the wrist. The lower arm of the subject was strapped in an arm rest while the subject held the handle of the WM. The axis of rotation of the WM was aligned with the axis of rotation of the wrist. The neutral angle was determined for each subject as an angle between flexion and extension which was comfortable for the subject. The lever of the WM is equipped with a force transducer to measure the torques exerted by the subject.

EEG was measured using 64 scalp electrodes, placed according to the 5 % electrode system (Oostenveld and Praamstra 2001) using a standard EEG cap with Ag/AgCl electrodes (actively shielded headcap by TMSi, Oldenzaal, the Netherlands). Electrode impedances were below 20 kΩ, and signal quality was monitored online. EMG was measured from the flexor carpi radialis $({\rm EMG}_{{\rm FCR}})$ using bipolar Ag/AgCl electrode pairs placed on the muscle belly. All physiological signals were sampled at 2,048 Hz (Refa system by TMSi, Oldenzaal, the Netherlands). The angle of the WM and the torque exerted on the lever were synchronously measured on a separate system at 2,048 Hz (Porti system by TMSi, Oldenzaal, the Netherlands) or via electrically isolated channels on the same amplifier as the physiological signals.

### Protocol

Subjects performed a motor task with the affected arm and with the non-affected arm. In the active motor task, subjects were instructed to exert a constant wrist flexion torque on the handle of the WM. Subjects were instructed to keep the exerted torque within a range of 1.8 ± 0.27 Nm. To aid the subjects in keeping a steady torque, subjects received visual feedback of the exerted and the target torque via a display. For the visualisation, the exerted torque was low-pass filtered online with a cut-off frequency below the bandwidth of the perturbation (third-order low-pass Butterworth, 2 Hz) to remove frequencies contained in the position perturbation.

All subjects performed the active motor task (active task) with the non-affected arm and attempted the active task with the affected arm. When a subject was unable to maintain a stable wrist flexion torque for at least 5 s with the affected wrist or when the subject was unable to return to the target torque once the exerted torque decreased, the subject performed the relax task. In the relax task, the subject held the handle of the WM without exerting a torque. Table 1 lists which task was performed with the affected arm for each subject. Subjects performed eight trials of 40 s. In case the online monitoring of the EEG revealed many eye blinks or activation of facial muscles, an extra trial was recorded. The affected arm trials were performed first. During the trials, the inactive hand lay in a comfortable relaxed position, generally on the lap of the subject. EMG was visually monitored online to control for mirror movements.

Six subjects (C.05, C.07, C.08, C.10, C.11, and C.13) were willing to perform both the active and relax task with the affected and/or non-affected arm to allow comparison between the active and relax task. Two subjects performed these extra trials in a separate measurement session. The subjects with poor motor function that performed the extra active motor tasks with the affected arm (C.05, C.07, and C.13) performed the extra active task with a lower target torque. The target torque was set such that muscle activation was seen in the EMG signal. Even with a low target torque, these subjects were unable to maintain a stable contraction; during the trials, subjects were motivated to keep attempting to exert torque and return to the target torque when the exerted torque decreased.

The position perturbation signal (Fig. 1, right side) consisted of a sum of sine waves (5, 9, 13, 17, 21, 25, and 29 Hz). The perturbation signal had a period of 1 s and a peak-to-peak amplitude of 1.7° (0.03 rad). The power of the sine waves decreased with frequency, giving the perturbation a flat velocity spectrum. The small amplitude of the perturbation allows the application of the perturbation also to subjects with increased wrist stiffness.

### Data analysis

Recorded signals were processed offline using MATLAB 2010b (the MathWorks, Inc., Natick, MA, USA). First, raw EEG signals were high-pass filtered (1 Hz, second-order Butterworth filter applied with zero phase shift) to remove baseline drift. Channels containing artefacts due to bad electrode contact were removed from the common average reference. EEG signals were low-pass filtered (70 Hz, second-order Butterworth applied with zero phase shift) and resampled to 1,024 Hz.

All signals were segmented in 1-s segments (1,024 samples)—the period of the perturbation—with 75 % overlap between segments. Segments were visually inspected, and segments that contained eye blinks or muscle activity were removed. The 50-Hz component was removed from each segment using the discrete Fourier transform (Oostenveld et al. 2011). EEG data were then referenced to a nearest neighbour Laplacian derivation.

Subsequent coherence analysis was performed on EEG channels overlying the left and right sensorimotor areas (SM). The left SM consists of FC1, FC3, FC5, C1, C3, C5, CP1, CP3, and CP5, and the right SM consists of the equivalent electrodes on the right hemisphere.

#### Estimation of PCC

All segments were transformed to the frequency domain using the fast Fourier transform. The power spectral density (PSD, $\varPhi _{xx}(f)$) and cross-spectral density (CSD, $\varPhi _{xy}(f)$) were estimated using

1 $$\varPhi _{xx}(f) = \frac{1}{N} \sum _{i=1}^{N} X_{i}^{*}(f) \cdot X_{i}(f)$$

and

2 $$\varPhi _{xy}(f) = \frac{1}{N} \sum _{i=1}^{N} X_{i}^{*}(f) \cdot Y_{i}(f)$$

respectively, where *X*_*i*_(*f*) and *Y*_*i*_(*f*) are the Fourier coefficients at frequency *f*, estimated from the *i*th data segment. The asterisk indicates the complex conjugate, and *N* is the total number of segments.

EEG channels were excluded from coherence analysis when the mean power in the frequency band between 25 and 49 Hz was larger than the mean power between 5 and 15 Hz. Channels with this power distribution were presumed to reflect mostly EMG activity. This method for marking channels with EMG activity is based on the method applied by Severens et al. (2012) to select EEG and EMG components in a blind source separation method. The presence of EMG activity obscures the detection of PCC as it severely decreases signal-to-noise ratio.

The (magnitude-squared) coherence (*C*_*xy*_(*f*)) between signals was calculated according to:

3 $$C_{xy}(f) = \frac{\vert \varPhi _{xy}(f)\vert ^{2}}{\varPhi _{xx}(f) \varPhi _{yy}(f)}.$$

Position-cortical coherence (PCC) was calculated between the position perturbation signal and each EEG channel and was only evaluated at the frequencies contained in the perturbation signal. The significance of coherence values was determined using the approximation of the confidence limit (CL) by Bortel and Sovka (2007). The confidence level was set to 0.99 (*α* = 0.01).

The presence of PCC across the sensorimotor area contralateral to the wrist perturbation (contra-SM) was evaluated by summing the number of frequencies where the PCC exceeds the 99 % CI per electrode and summing across the contra-SM. This number was expressed as a percentage of the total number of frequency bins on the contra-SM (i.e. number of stimulus frequencies times the number of electrodes in the contra-SM). Amplitude of significant PCC was evaluated by the mean significant PCC over the contra-SM.

Lateralisation of PCC was quantified by the lateralisation index (*L*):

4 $$L = {\rm log}_{10}({\rm PCC}_{\text{contra-SM}}) - {\rm log}_{10}({\rm PCC}_{\text {ipsi-SM}})$$

where ${\rm PCC}_{\text{contra-SM}}$ and ${\rm PCC}_{\text{ipsi-SM}}$ are the mean PCC amplitudes over all frequencies and all electrodes in the contra-SM and ipsilateral SM (ipsi-SM), respectively. Note that in the lateralisation index, no distinction is made between the presence and amplitude of significant PCC. When *L* > 0, the PCC is more lateralised towards the contra-SM. L < 0 indicates that PCC is more lateralised towards the ipsi-SM.

The nonparametric Wilcoxon rank-sum test was used to compare the presence and amplitude of significant PCC at the contra-SM and the lateralisation index between the good function and poor function subjects. Paired *t* tests were used to compare contra-SM PCC and lateralisation index in response to affected wrist perturbation and non-affected wrist perturbation within one subject. Amplitude of significant PCC was log_10_-transformed prior to statistical analysis.

## Results

All good function subjects had normal sensory function on all test items according to the EmNSA scale (see Table 1). In the poor function group, C.07 had a reduced sensory function affected arm at the time of the first measurement session (11 days post-stroke). C.07 had a reduced tactile sensation in the hand and fingers and a reduced ability to discriminate between sharp and blunt tactile stimuli in the whole arm including hand and fingers. In addition, C.13 had a reduced sensory function in the affected arm: a reduced ability to discriminate between sharp and blunt stimuli across the whole arm including hand and fingers and a reduced proprioceptive sense in the fingers, wrist, and elbow.

In all eleven subjects, EEG on one or more electrodes in the left and right SM was excluded due to poor signal quality. In nine subjects, this concerned one or more of the most temporal electrodes (FC5, FC6, C5, C6, CP5, and CP6). In two subjects, also electrodes other than the most temporal ones were excluded. For C.06, electrodes FC3, C3, and CP3 were excluded in addition to the most temporal electrodes (FC5, FC6, C5, C6, CP5, and CP6). For C.07, electrodes C3 and FC3 were excluded in addition to the most temporal electrodes (FC5, FC6, C5, and C6).

### Presence and amplitude of significant PCC in the contralateral sensorimotor area

**Fig. 2:**
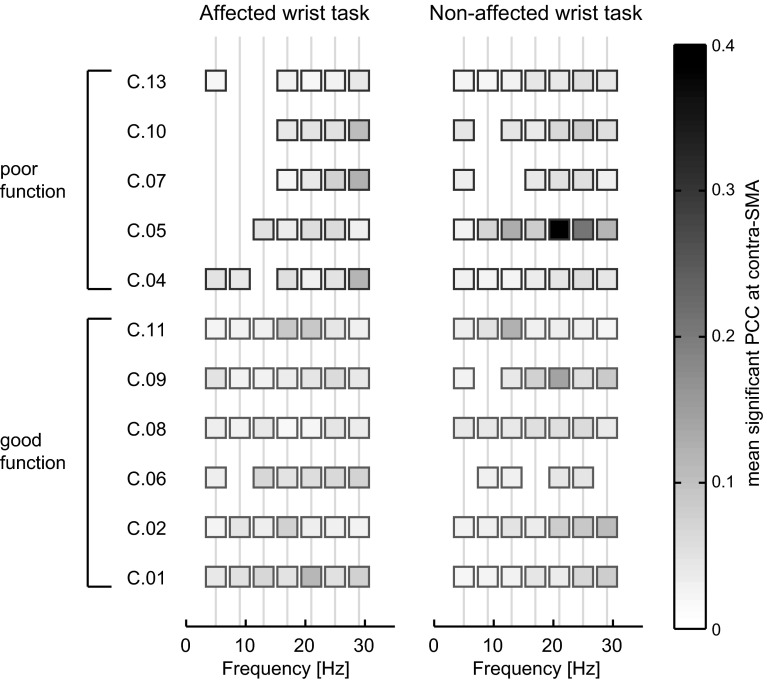
Mean significant PCC at the contra-SM per stimulus frequency. *Left* affected wrist task, *right* non-affected wrist task. *Grey vertical lines* indicate the stimulus frequencies, a *square* indicates that the PCC was significantly larger than zero at that stimulus frequency on at least one electrode in the contra-SM

All subjects presented significant PCC at the contra-SM on at least three stimulus frequencies in the affected wrist task and in the non-affected wrist task (see Fig. 2). Five of the six subjects in the good function group had significant contra-SM PCC during the affected wrist task on all stimulus frequencies, while none of the subjects in the poor function group had significant contra-SM PCC on all stimulus frequencies during the affected wrist task. The poor function subjects all presented significant PCC on the highest stimulus frequencies (17, 21, 25, and 19 Hz) in the affected wrist task.

**Fig. 3:**
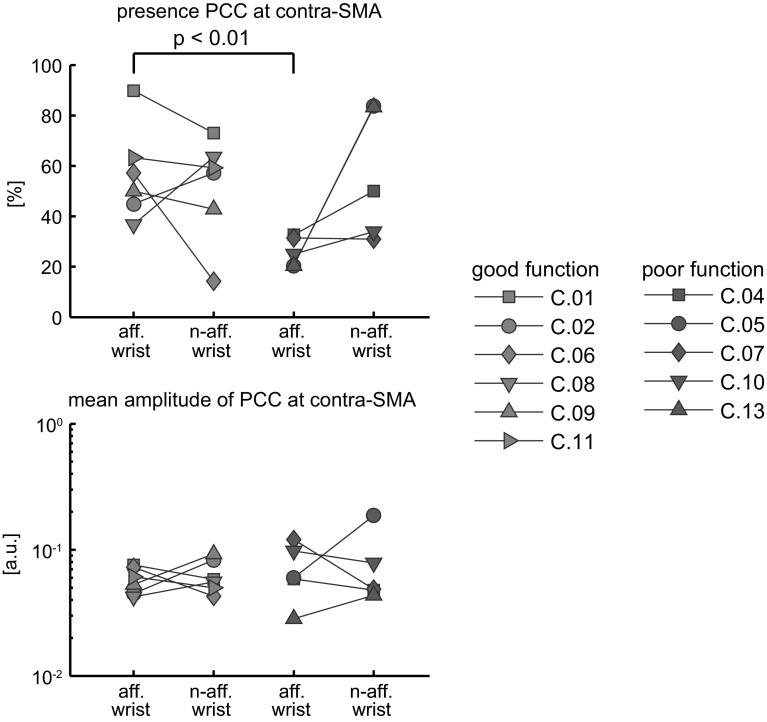
Presence and amplitude of significant PCC at the contra-SM in the affected wrist task (aff. wrist) and non-affected task (n-aff. wrist). *Upper panel* Presence of PCC expressed as a percentage of the total number of frequency bins on the contra-SM. *Lower panel* Mean significant PCC over the contra-SM and all stimulus frequencies in arbitrary units (a.u.)

The presence of significant PCC at the contra-SM varied between affected and non-affected wrist tasks and between subjects (Fig. 3). Poor function subjects tended to have a lower presence of contra-SM PCC in the affected wrist task compared with the non-affected wrist task but the difference did not reach statistical significance. The difference in the presence of contra-SM PCC in the affected wrist tasks between poor and good function subjects was significant (Wilcoxon rank-sum test: *p* < 0.01). The average difference between the subject groups was 31 %.

The mean significant amplitude of PCC on the contra-SM varied between subjects and between affected and non-affected wrist tasks (Fig. 3). There was no significant difference in mean PCC amplitude at contra-SM between good and poor function subjects, not in the affected wrist task and not in the non-affected wrist task. Neither was there a significant difference between contra-SM PCC amplitudes in affected and non-affected wrist tasks within subjects.

### Comparison of PCC in active and relaxed tasks

**Fig. 4:**
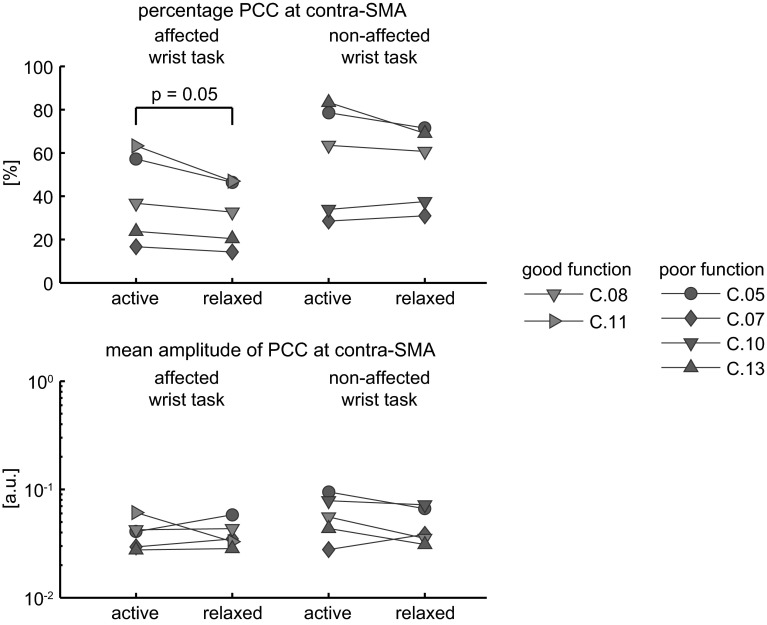
Comparison of the presence and magnitude of significant PCC between an active and a relaxed task. *Upper panel* Presence of PCC expressed as a percentage of the total number of frequency bins across the contra-SM. *Lower panel* Mean significant PCC over the contra-SM in arbitrary units (a.u.). C.10 performed both tasks only with the non-affected wrist, and C.11 performed both tasks only with the affected wrist

The difference in tasks performed with the affected wrist by the subjects with poor function and those with good motor function (relax and active motor tasks, respectively) could be a confounding factor. Therefore, five subjects performed an extra active or relax task with the affected and/or non-affected wrist to enable comparison of the presence and mean significant amplitude of contra-SM PCC between the active and relax tasks. Results are summarised in Fig. 4. The presence of contra-SM PCC in the affected wrist tasks tended to be lower in the relax task compared with the active task. This difference was on the limit of significance (paired *t* test: *p* = 0.05). The average difference in the presence of contra-SM PCC was 7.4 %. In the non-affected wrist tasks, the difference was not significant. There were no significant differences in mean significant contra-SM PCC amplitude between active and relaxed tasks.

### Lateralisation of PCC

**Fig. 5:**
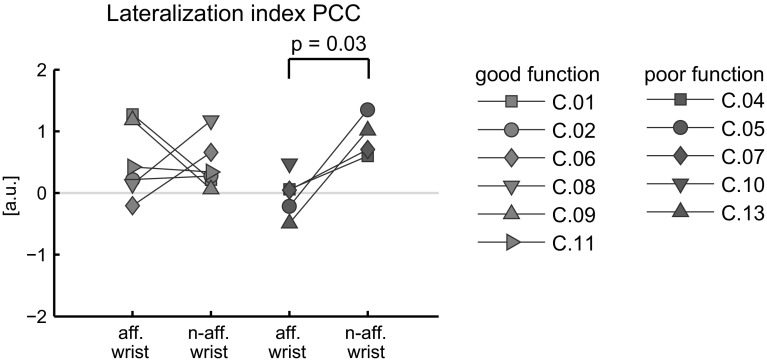
Lateralisation index for affected and non-affected wrist tasks (aff. wrist and n-aff. wrist, respectively) in arbitrary units (a.u.). *L* > 0 indicates that PCC is more lateralised towards the contra-SM compared with the ipsi-SM. *L* < 0 indicates that PCC is more lateralised towards the ipsi-SM

In ten subjects, PCC was significantly larger than zero on at least one electrode on the ipsi-SM during both the affected and the non-affected wrist task. Only C.10 did not show significant PCC on the ipsi-SM during the non-affected wrist task, and thus, the lateralisation index could not be determined in this case. In the non-affected wrist task, the lateralisation index was larger than zero in all subacute subjects, showing that PCC was lateralised more towards the contra-SM (see Fig. 5).

In the poor function group, the lateralisation index was significantly lower during the affected wrist task compared with the non-affected wrist task (paired *t* test: *p* = 0.03). This indicates that in the poor function group, PCC is distributed more evenly between the lesioned and non-lesioned hemisphere during the affected wrist task, while during the non-affected wrist task PCC is more lateralised to the (non-lesioned) contra-SM.

Three subjects (C.06, C.05, and C.13) had a negative lateralisation index during the affected wrist task. This indicates that the PCC is more lateralised towards the ipsi-SM.

## Discussion

In this study, we evaluated position-cortical coherence (PCC) in eleven subacute stroke subjects. Position-cortical coherence is the coherence between a joint position perturbation and the EEG during an isotonic motor task. Because the position perturbation acts as an independent external excitation of the proprioceptive system, PCC represents unidirectional connectivity, i.e. information transfer, across the afferent pathways (Campfens et al. 2013). All stroke subjects presented PCC at the contralateral sensorimotor area (contra-SM) during an affected and non-affected wrist angle perturbation. The presence of contra-SM PCC during an affected wrist perturbation was lower in poor motor function stroke subjects than in good motor function stroke subjects. The presence of contra-SM PCC differed between subjects with good and poor motor function; the amplitude of significant contra-SM PCC did not differ between these groups. This implies that when contra-SM PCC is significantly larger than zero, the amplitude does not contain extra information. The reduced presence of PCC in the ipsilesional hemisphere of poor function subjects shows that these subjects have not only poor functioning efferent motor pathways, but a reduced integrity of their afferent sensory pathways as well. This agrees with the notion that motor control takes place in a closed loop where sensory feedback is crucial for generating proper motor commands.

From the reduced presence of PCC, it cannot directly be determined whether subjects with poor motor function showed contra-SM PCC on less frequencies or on less electrodes. Damage to the sensorimotor cortex due to a large cortical stroke can result in a reduction in the size of the sensorimotor cortex and thus PCC on less electrodes. However, none of the poor function subjects presented significant PCC on the lowest stimulus frequencies in the affected wrist task (Fig. 2). This indicates that a reduced number of frequencies where PCC is present is a large contributing factor to the reduced presence of PCC in the poor function subjects. Future studies combining PCC measurements with detailed imaging of the cortex should correlate the presence of PCC to specific (cortical) damage.

In most data sets, electrodes had to be excluded because the EEG signals were contaminated by muscle activity. As a result, we may have missed significant PCC. For future studies, it is advised to consider the use of artefact removal algorithms to remove muscle activity artefacts from the EEG. A potentially suitable algorithm based on blind source separation was presented by De Vos et al. (2010) for the removal of tonic muscle activity from EEG recorded during spoken language.

In the group of subacute stroke subjects included in this study, the subjects with good and poor motor function had very distinct ranges of FM-UE scores. Due to this sharp distinction, we cannot correlate the PCC measures with the FM-UE score. In a larger population of stroke subjects, that exhibit a more continuous distribution of FM-UE scores, it would be possible to perform correlation analysis on the presence of contra-SM PCC and motor function score to find out whether there is a continuous relation between motor function and afferent pathway integrity.

Although the presence of contra-SM PCC in the affected wrist task was lower in the poor function group, most subjects in the poor function group had a normal sensory function according to the EmNSA (Stolk-Hornsveld et al. 2006). Subject C.13 had a reduced proprioceptive sense of the fingers, wrist, and elbow but a presence of PCC similar to the other subject in the group. This implies that sensory feedback related to the perturbation from the muscle spindles and Golgi tendon organs does reach the cortex. Possibly the sensory feedback is processed in a different, less effective, manner at the cortical level, resulting in the reduced sensory function according to the clinical assessment of this subject. However, as the other subjects with poor motor function had normal sensory function according to the clinical assessment, the lower presence of PCC in the poor function group could suggest that sensory deficits in this group were not found with the clinical assessment. Clinical scaling of sensory function has been shown to be unreliable (Lincoln et al. 1991). Although progress has been made to obtain more reliable clinical scoring of sensory function (Stolk-Hornsveld et al. 2006), especially impairment of proprioception is often overlooked (Dukelow et al. 2010). Dukelow et al. (2010) introduced a method for the assessment of proprioceptive function using robotics. Such robotic assessment of sensory function could very well be combined with an evaluation of PCC to enable a more quantitative evaluation of the relation between PCC and proprioceptive function.

Subjects with poor motor function were not able to generate a steady wrist flexion torque, and these subjects performed the relax task. Also during the relax task, subjects showed significant contra-SM PCC. In the subjects that performed additional active and relax tasks for comparison, the presence of contra-SM PCC did not differ between active and relax tasks performed with the non-affected wrist. This implies that the cortical response to the position perturbation is similar during active and relax tasks. However, when performed with the affected wrist the presence of PCC was 7 % smaller during a relax task. This difference between active and relax tasks is not sufficient to explain the difference in the presence of PCC found between the subjects with good and poor motor function (30 %). Although the difference between active and relax tasks is small or absent, we advise to let subjects relax in future studies to fully avoid biased results due to differences in ability to perform motor tasks.

The frequency-dependent likelihood of finding significant contra-SM PCC during the affected wrist task in the poor function group indicates that there is a frequency dependency of the signal-to-noise ratio in the ipsilesional EEG. Either the EEG contains more contributions of sources other than the position perturbation at the lowest stimulus frequencies (increased noise at lowest stimulus frequencies) or the contribution of the position perturbation to the EEG is higher at the highest stimulus frequencies (increased signal at the highest stimulus frequencies). The perturbation signal was designed such that it had a flat velocity spectrum. As a result, the position- and velocity-sensitive muscle spindles were excited with decreasing and equal power at each frequency, respectively. Higher sensitivity of muscle spindles to higher velocities or excitation of Golgi tendon organs could still result in a higher output of the sensory pathways at the higher stimulus frequencies, thus increasing the likelihood of finding PCC at these frequencies. An alternative explanation for an increased signal at the highest stimulus frequencies could be that these stimulus frequencies are transmitted more efficiently along the afferent pathways. The higher stimulus frequencies lie in the beta band (15–30 Hz). During an isotonic and isometric motor task, coherence between EEG and EMG is typically found in the beta band, indicating that oscillations in this frequency band are already being transmitted along the afferent pathways.

Using the lateralisation index of PCC, we found that three of the eleven subjects (two poor function and one good function) had PCC predominantly localised in the ipsi-SM during the affected wrist task. It is known from several studies that after stroke, there can be a disbalance between the lesioned and the non-lesioned hemisphere. This results in abnormal activation of the ipsilateral sensorimotor cortex during motor tasks with the affected hand and is especially seen in stroke survivors with poor recovery of motor function (Ward 2003; Serrien et al. 2004). During motor tasks with the affected side, the increased activity of the ipsilateral sensorimotor cortex is seen as a sign of increased motor output of the ipsilateral sensorimotor cortex. Our results in lateralisation of PCC indicate that sensory input from the affected wrist elicits response in the ipsilateral sensorimotor cortex and that this response can even be stronger than in the contralateral sensorimotor cortex. Increased activation of the ipsilateral sensorimotor cortex can thus also indicate increased sensory input to this area during an affected arm motor task. Further research is required to establish whether this relates to recovery of motor function.

About two-third of the stroke survivors with initial hemiplegia do not regain dexterity and remain impaired despite rehabilitation therapy (Kwakkel et al. 2003; Dobkin 2005). The ability to extend the fingers and abduct the shoulder at 72 h post-stroke gives a strong indication of an individual’s ability to recover motor function (Nijland et al. 2010). Nevertheless, 25 % of the stroke survivors with an initial poor prognosis does regain dexterity. It is important to identify individuals in this so-called crossover group since they will most likely benefit most from early applied intensive rehabilitation training. Potentially, objective assessment of connectivity between cortex and periphery could aid in identifying individuals in the crossover group. Position-cortical coherence may be more suitable for this purpose because it has important advantages over CMC, which is the traditional measure of corticomuscular connectivity (Campfens et al. 2013). While CMC cannot be found in all subjects, all subjects do present PCC. Furthermore, PCC can be measured even in subjects that lack the ability to voluntarily generate muscle contraction, while muscle activation measurable by EMG is required for the estimation of CMC. In a larger longitudinal study design, it should be evaluated whether PCC, as an objective measure of sensory pathway integrity, provides additional information that allows separation between the good prognosis, poor prognosis, and crossover group.

### Conclusion

This study shows that subacute stroke subjects present PCC, indicating that afferent sensory information arrives at the cortex. Position-cortical coherence can even be detected in subjects with very poor motor function, who are unable to generate voluntary force. The presence of PCC on the sensorimotor area contralateral to the affected wrist is lower in poor function subjects compared with good function subjects. This shows that afferent pathways integrity and sensorimotor integration are affected in stroke survivors with poor motor function. In addition, in subjects with poor motor function, the lateralisation index showed that PCC is distributed more evenly between the lesioned and non-lesioned hemisphere during affected wrist perturbations than during non-affected wrist perturbations. Position-cortical coherence provides an objective measure of sensory pathway information transfer and processing after stroke and may provide additional insight into mechanisms of recovery of motor function after stroke.
